# Benefits of Coiling Aorto-Pulmonary Collaterals in Children with Complex Congenital Heart Diseases

**DOI:** 10.1007/s00246-025-03946-x

**Published:** 2025-07-15

**Authors:** Ashley Molloy, Neil Tailor, Katherine Hunter, Umar Boston, Shiva Sathanandam, Shyam Sathanandam

**Affiliations:** 1 Division of Pediatric Cardiology, LeBonheur Children’s Hospital, University of Tennessee Health Science Center, 848 Adams Avenue, Memphis, TN 38103 USA; 2 Heart Center, Division of Cardiac Surgery, Children’s Hospital of the King’s Daughters, Norfolk, VA USA; 3 James Cook University Hospital, Middlesbrough, UK

**Keywords:** Aortopulmonary collateral vessels, Fontan palliation, Heart transplant, Coil embolization, Cardiac catheterization

## Abstract

This study investigates the benefits of coiling aortopulmonary collaterals (APCs) before Fontan completion and prior to heart transplantation due to failed Fontan. The advantages of APC coiling in these situations remain unclear. Outcomes were compared between those undergoing the Fontan operation between June 2013 and December 2015, who did not undergo coiling of APCs, and those between January 2016 and May 2022, when aggressive coiling of APCs was performed. The 1-year post-transplant survival was compared for patients from Memphis, TN, where aggressive APC coiling was performed before transplantation, to a previously published report from St. Louis, MO, where APCs were actively coiled and an earlier era when they were not. The 44 Fontan patients with prior APC coiling were compared to 22 patients matched for age, diagnosis, and hemodynamics. The chest tube output (22.6 ± 6.1 vs. 41.8 ± 8.2 mL/kg; *P* < 0.001), the chest tube duration (5.1 ± 1.1 vs. 10.3 ± 4.5 days; *P* < 0.001), and the hospital length of stay (9.9 ± 1.7 vs. 27.4 ± 6.2 days; *P* < 0.001) were significantly lower for those who had APC coiling compared to those who did not. In St. Louis, MO, when APCs were not coiled before transplantation (*N* = 27), the 1-year survival rate was 66%, which improved to 85% (*N* = 20) in the era of APC coiling. In the Memphis experience (*N* = 25) with aggressive APC coiling, the 1-year survival rate was 92% (*P* = 0.018). APC coiling before Fontan completion decreases chest tube output and hospitalization days. It may also improve the 1-year survival rate after heart transplantation for children with failed Fontan.

## Introduction

Children with complex congenital heart diseases (CHD) with single ventricle physiology who have undergone cavopulmonary connections often have significant aortopulmonary arterial collaterals (APCs). However, the significance of APCs on clinical outcomes is not well understood. While some studies report APCs with increased morbidity after Fontan completion [1–5], others have found no such associations [6–9]. Therefore, there is a wide variation in the practice of transcatheter embolization of APCs before the completion of Fontan surgery [10]. Some centers believe in routine embolization of APCs before the Fontan operation, whereas others do not care to. Some prefer selective embolization based on a combination of angiographic and hemodynamic assessment. Due to the lack of outcome studies [10], none of these strategies are based on objective data. There is no comprehensive published data exploring whether effective embolization of APCs before completion of Fontan improves outcomes available.

Magnetic resonance imaging (MRI) can quantify APC flow under physiological conditions [11, 12]. Previous studies have confirmed that APC flow is substantial in most single ventricle patients after superior cavopulmonary connections [11–14]. Collaterals accounted for 30% to 40% of aortic flow and 50% to 60% of pulmonary flow [11, 12]. Only one study [5] has utilized MRI quantification of APC flow and demonstrated a significant linear association between APC flow and duration of hospitalization and chest tube duration. At our institution, since June 2013, we have performed routine cardiac MRIs and quantified APC burden before the Fontan operation. From January 2016 onwards, we instituted a protocol to embark on aggressive coiling of APCs during the pre-Fontan cardiac catheterization procedure if the APCs' contribution was ≥ 30% of aortic and/or ≥ 50% of pulmonary blood flow.

Most Fontan circulation has the potential to fail eventually, leading to severe morbidity such as protein-losing enteropathy and plastic bronchitis, among others [15]. Inevitably, many patients require heart transplantation at some point as the next palliation [15]. However, the post-transplant survival for Fontan patients is significantly lower than that for patients with cardiomyopathy or other CHDs [16–21]. Most report a 1-year survival for Fontan patients undergoing transplantation between 65 and 80% [16–21]. Several factors have been associated with worse outcomes for Fontan patients after heart transplantation, but none are predictive of decreased survival [20, 22, 23]. Previously, APCs were never considered a risk factor for graft failure until one study described that early detection and treatment of APCs by coil embolization is necessary to improve the post-transplant course in these complex patients [24]. They went on to suggest that APCs should be considered a cause of early donor heart failure in children following heart transplantation.

The group in St. Louis, MO, also discovered that Fontan patients with more significant APC burden had early graft failure [25]. In 2009, their team implemented an aggressive catheter-based APC embolization strategy before transplantation and compared the survival of these patients to an earlier era. The survival of Fontan patients undergoing heart transplantation improved from 63 to 90% after an aggressive APC coiling strategy was utilized [25]. At our institute, we started transplantation on single ventricle patients in November 2016. We have adopted an aggressive approach to coil APCs as soon as a patient is listed for transplantation and perform multiple catheterization procedures with further APC coiling, if necessary, until the patient undergoes transplantation. This article describes the outcomes of our aggressive approach to APC coiling before Fontan completion as well as before heart transplantation in children with Fontan failure.

## Methods

### Patient Selection

This study included two separate groups of patients. The first study included children undergoing Fontan palliation for single ventricle CHD between June 2013 and May 2022. They underwent a cardiac MRI before the pre-Fontan cardiac catheterization as a routine practice. Often, this MRI was performed within the same day as the cardiac catheterization under the same anesthesia encounter with results of the APC burden from the MRI being communicated directly with the interventionalist prior to the procedure. The APC burden was considered significant if it accounted for ≥ 30% of aortic and/or ≥ 50% of pulmonary venous flow. We adopted an aggressive APC coiling approach from January 2016 onwards if the APC burden based on the MRI was significant. This accounted for two cohorts of patients, all of whom had MRI-defined significant APC burden: one between June 2013 and December 2015, who did not undergo coiling of APCs, and the other between January 2016 and May 2022, who underwent aggressive coiling of APCs.

The second study involved children who underwent heart transplantation due to Fontan failure between November 2016 and May 2022. All patients received aggressive APC coiling, often multiple times, before their transplant. Since no historical controls were available from our institute, the results were compared to those published in a St. Louis, MO [25] study. They examined patient outcomes across two distinct eras: the first era, from 1995 to 2008, during which coiling of APCs was performed infrequently, and the second era, from 2009 to 2014, when APCs were actively surveyed and coiled before transplantation.

### Outcome Measures

For Fontan patients, the outcomes compared between those who underwent APC coiling and those who did not include the chest tube output on the day of the Fontan operation (postoperative day #0), the duration of chest tube output, the fluid balance on postoperative days (POD) #0 and #1, as well as the hospital length of stay. Patients were matched based on age at Fontan, weight, gender, and underlying anatomy. They were also matched for hemodynamics based on the pre-Fontan catheterization. Only patients with pulmonary vascular resistance (PVR) ≤ 2 WU*m^2^ and ventricular end-diastolic pressure (VEDP) ≤ 10 mmHg were included.

The primary outcome for the transplant patients with failed Fontan was 1-year survival among three groups of patients. The patients from our institute in Memphis, TN, who were transplanted for Fontan failures between November 2016 and May 2022, were compared against patients from the two separate eras in St. Louis, MO. Other measures between the three groups included comparing the number of patients with ≥ 5 APCs coiled before transplantation and the number of embolization events for each patient and the entire group.

### Coiling Protocol for APCs

**Fig. 1:**
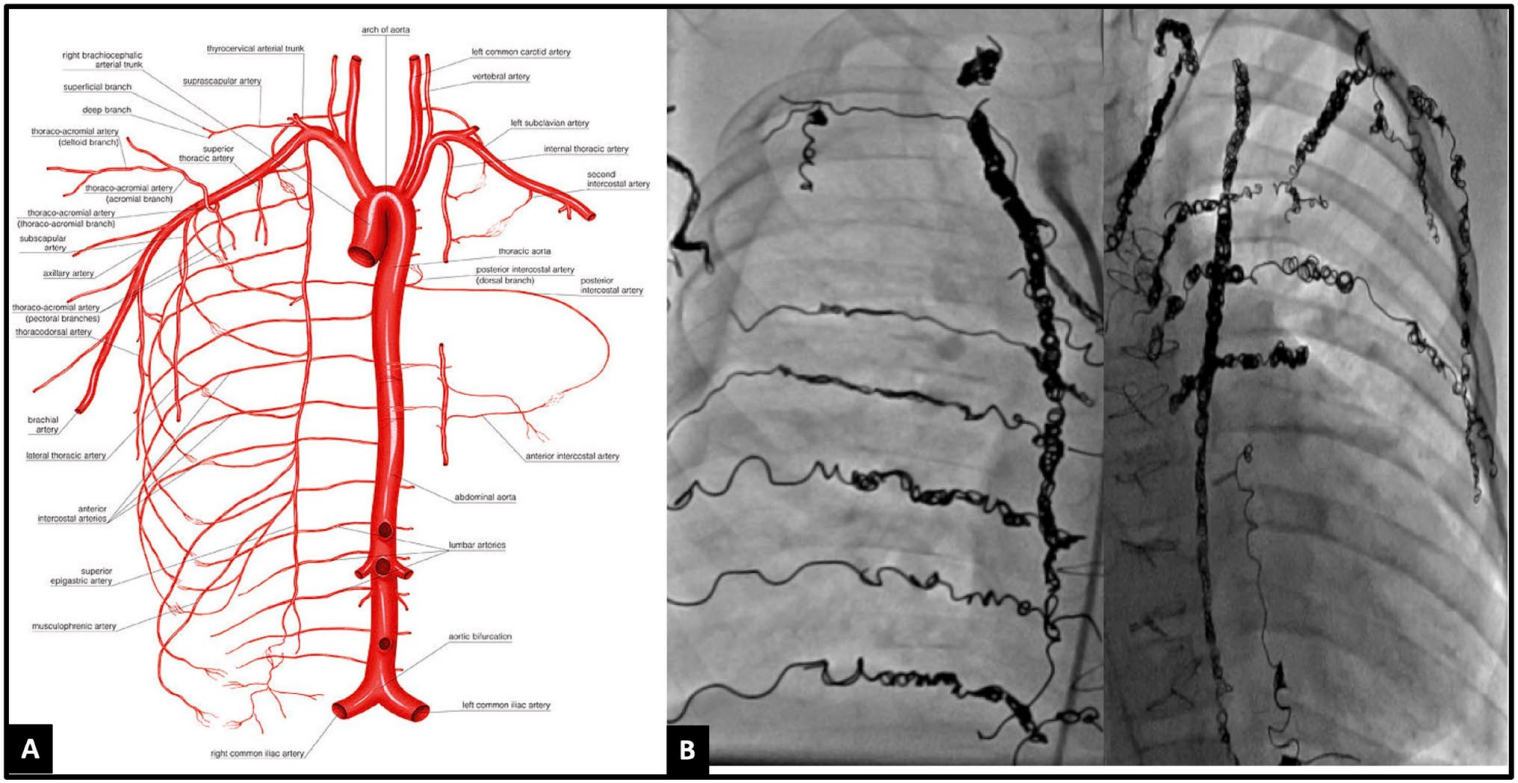
**A** A network of vertical and horizontal arteries encircles the thoracic cavity, serving as sources for APCs. **B** An example of aggressive APC coiling in this 13-year-old child with hypoplastic left heart syndrome post-Fontan, listed for heart transplantation due to protein-losing enteropathy. These were performed over 5 different catheterization procedures

We adopted an aggressive APC coiling strategy that included active surveillance for APCs during pre-Fontan or pre-transplant cardiac catheterizations. Figure 1A describes the blood vessels where APCs could arise, while Fig. 1B demonstrates a pre-transplant patient who underwent extensive APC coiling over three different catheterization procedures. It is crucial to survey APCs by selectively engaging the catheter into the common blood vessels from which they arise and performing digital subtraction angiography (DSA). A vertical and horizontal network of arteries surrounds the thoracic cavity from which APCs can arise. Our protocol involves occluding the entire network grid so that new APCs do not develop from anastomoses with other arteries.

**Fig. 2:**
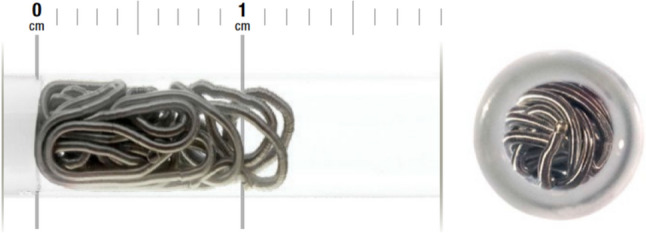
Penumbra packing coil

The Penumbra embolization platform (Penumbra Inc, Alameda, CA), which includes Ruby® Coils, POD®, Packing Coils, and LP Coils, provides significant advantages in managing the complex vascular anatomy often found in pediatric patients. We prefer Packing Coils (or LP Packing Coils) alongside a framing coil (Ruby Soft or Ruby LP) as a backstop. Each Penumbra coil technology is characterized by softness and volume (Fig. 2). The enhanced softness of these coils enables mechanical vessel occlusion without the need for fibers, reducing their reliance on thrombus formation.

### Statistical Analysis

Categorical data are reported as counts and percentages, and continuous data are reported as medians and ranges to present the minimum and maximum values or as means and standard deviations (SD). The quantile regression analysis was used to analyze the relationship between variables across different quantiles of the data obtained from the Fontan patients, except for the outcomes for which the *t*-test for independent samples was utilized. The one-way ANOVA test was applied to compare the three groups of post-transplantation patients. All statistical analyses were defined by a two-tailed *P* < 0.05 for significance. Statistical analysis was performed using IBM SPSS Statistics (version 27.0, IBM, Armonk, NY).

## Results

### Comparison of Outcomes for Fontan Patients With or Without APC Coiling

**Fig. 3:**
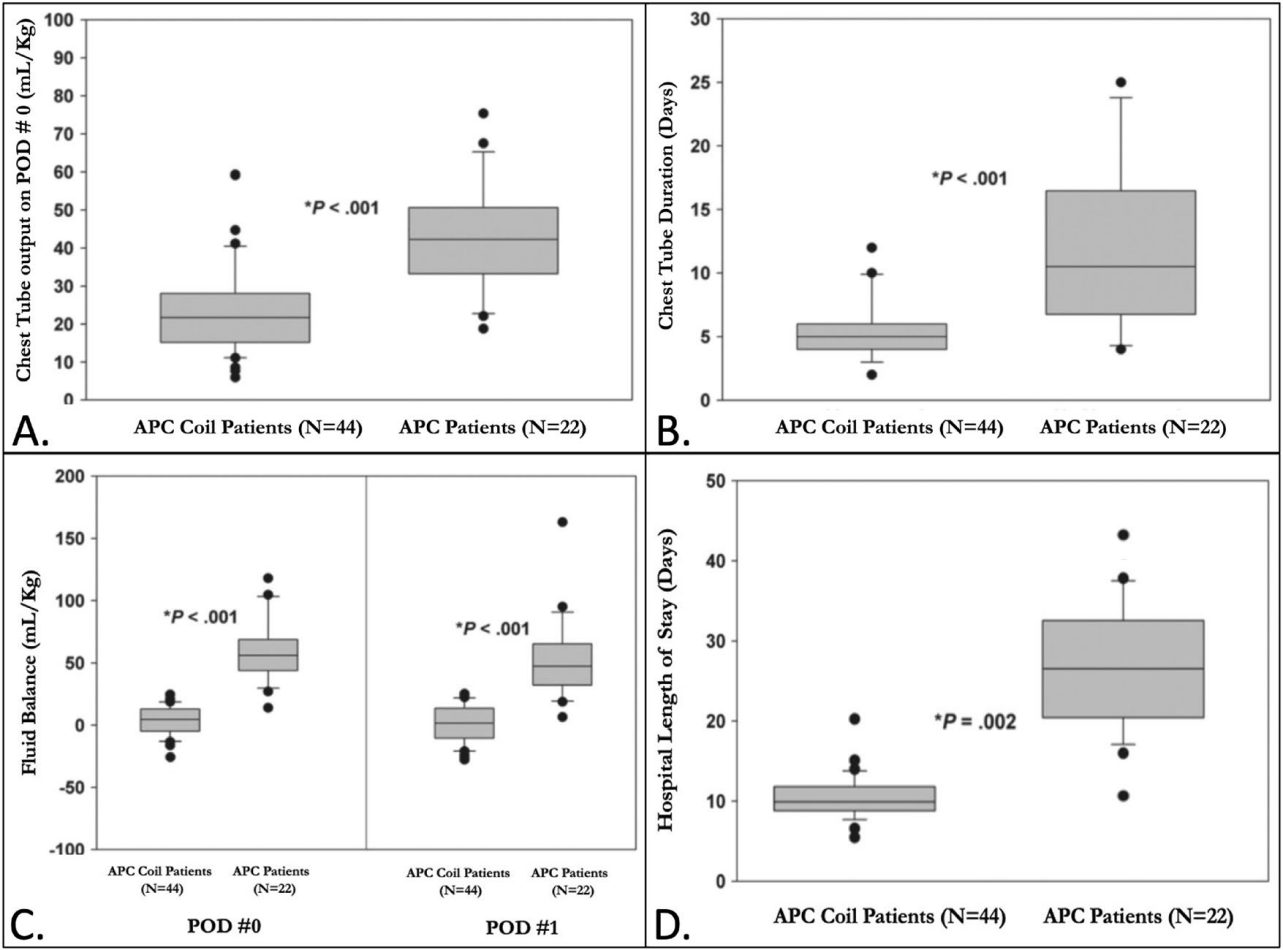
Comparison of outcomes following the Fontan operation between patients with APC coiling and those without: **A** The volume of chest tube drainage on POD # 0 is significantly lower for patients who underwent prior APC coiling. **B** The duration of chest tube necessity is significantly shorter for patients who underwent prior APC coiling. **C** The patients who underwent prior APC coiling had better fluid balance on POD # 0 and POD # 1 compared to those who did not have APC coiling. **D** The hospital length of stay was shorter for patients who underwent prior APC coiling

Based on the inclusion criteria, 44 patients who underwent APC coiling were compared against 22 matched patients who did not. The demographics of these patients are summarized in Table 1. All patients had APC burden accounting for ≥ 30% of aortic and/or ≥ 50% of pulmonary venous flow on MRI. Six patients from both groups were excluded since they had PVR > 2 WU*m^2^ or VEDP > 10 mmHg. The pre-Fontan hemodynamics and operative variables are also described in Table 1. There were no significant differences between the patients in either group regarding age, gender, weight, or the underlying anatomical substrate at the time of the Fontan operation, nor were there differences in pre-operative hemodynamics or the operative variables (Table 1). However, significant outcome differences were observed (Table 1 and Fig. 3). The chest tube output on POD #0 (41.8 ± 8.2 vs. 22.6 ± 6.1 mL/kg; *P* < 0.001) and the duration of the chest tube (10.3 ± 4.5 vs. 5.1 ± 1.1 days; *P* < 0.001) were significantly higher for those who did not have APC coiling compared to those whose APCs were occluded before the Fontan operation. The mean hospital length of stay was almost three times shorter when APCs were coiled than when they were not (9.9 ± 1.7 vs. 27.4 ± 6.2 days; *P* < 0.001).

**Table 1:** Patient characteristics and outcomes of patients undergoing Fontan completion with and without APC coiling

	APC coil patients (*N* = 44)	APC patients (*N* = 22)	*P* value
Demographics			
Age (months)	30 (21–48)	32 (24–54)	0.34
Male (%)	22 (50%)	11 (50%)	–
Weight (kg)	12.6 (9.5–15.1)	13.2 (10.2–15.5)	0.78
RV morphology (%)	26 (59%)	12 (55%)	0.77
HLHS sub-types (%)	24 (55%)	11 (50%)	0.56
Pre-Fontan hemodynamics			
Mean PA pressure (mmHg)	12 (10–13)	12 (11–13)	0.91
PVR (WU*m^2^)	1.45 (1.03–1.98)	1.7 (1.28–1.80)	0.36
VEDP (mmHg)	9 (6–10)	8 (7–9)	0.52
Operative variables			
Bypass time (min)	80 (61–98)	80 (64–102)	0.92
X-Clamp time (min)	0 (0–0)	0 (0–15)	0.08
Fenestration (%)	3 (6.8%)	3 (13.6%)	0.22
Post-Fontan outcomes			
Survival (%)	44 (100%)	22 (100%)	–
Chest tube output on POD#0 (mL/kg)	22.6 ± 6.1	41.8 ± 8.2	< 0.001*
Chest tube duration (days)	5.1 ± 1.1	10.3 ± 4.5	< 0.001*
Fluid balance on POD#0	7.0 ± 9.2	58.4 ± 16.8	< 0.001*
Fluid balance on POD#1	1.7 ± 10.6	49.7 ± 18.2	< 0.001*
Hospital stay (days)	9.9 ± 1.7	27.4 ± 6.2	< 0.001*

*Statistically significant

### Comparison of Outcomes of Children Undergoing Heart Transplantation for Fontan Failure With or Without APC Coiling

**Fig. 4:**
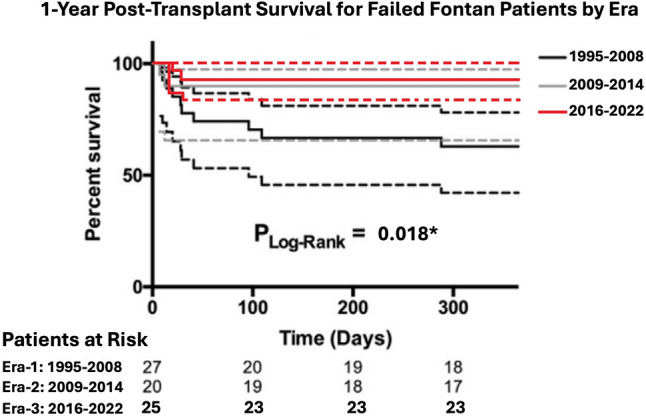
1-Year post-transplant survival for failed Fontan patients by era

The previously published paper from St. Louis, MO [25], found that in the era when APCs were not actively coiled before transplantation (*N* = 27), the 1-year survival rate was 66%, which improved to 85% (*N* = 20) in the era of APC coiling (patients with ≥ 1 APCs coiled = 22% vs. 85%). In our experience (*N* = 25) with aggressive APC coiling, the 1-year survival rate was 92% (*P* = 0.018). This strategy was aggressive because all patients had ≥ 5 APCs coiled. There were 86 catheterizations for coil embolization of APCs for the 25 patients, with a median of 3.4 procedures per patient, significantly higher than those from St. Louis, MO, where patients averaged 1.5 coiling procedures each. The comparison between the three groups is summarized in Table 2 and Fig. 4.

**Table 2:** Improved post-transplant survival for failed Fontan with aggressive APC coiling

Aortopulmonary collaterals	St. Louis experience		Memphis experience (2016–2022) *N* = 25	*P* value
	(1995–2008)	(2009–2014)		
	*N* = 27	*N* = 20		
Presence of APCs (%)	14 (52%)	17 (85%)	25 (100%)	< 0.001*
Patients with ≥ 1 APCs coiled (%)	6 (22%)	17 (85%)	25 (100%)	< 0.001*
Patients with ≥ 5 APCs coiled (%)	0 (0%)	5 (25%)	25 (100%)	< 0.001*
Total coiling event per group (%)	7 (26%)	30 (150%)	86 (344%)	< 0.001*
Coiling events per patient	0.3 (0–2)	1.5 (0–7)	3.4 (1–6)	< 0.001*
1-year survival (%)	18 (66%)	17 (85%)	23 (92%)	0.018*

*Statistically significant

## Discussion

Ambiguity remains surrounding the necessity of APC coiling before the Fontan operation. This study analyzed two patient groups: one that did not receive APC coiling before Fontan completion and another that underwent aggressive APC coiling informed by MRI flow quantification. While both cohorts were similar in age at the time of Fontan completion, underlying diagnosis, and hemodynamic status, individuals with previous APC coiling experienced significantly reduced chest tube drainage and fewer days of postoperative hospitalization. This leads to lower morbidity and reduced hospital costs. These findings align with Glatz et al. [5], who reached the same conclusions when comparing their patients pre-Fontan cardiac MRI findings to their acute post-Fontan outcomes.

The debate continues regarding the necessity of detecting and treating APCs through coil embolization to enhance post-transplant outcomes and survival in children with failed Fontan circulation. This study and the one from St. Louis, MO [25], show an increase in 1-year post-transplant survival with proactive APC coiling before transplantation. However, attributing this survival benefit solely to APC coiling is abstruse due to the patients spanning three different decades. Nonetheless, removing the APCs and alleviating the volume burden on the newly transplanted heart might have contributed to better survival rates. One factor behind the numerous coiling procedures in our group was the lengthy wait times for some patients, which resulted in the formation of new APCs between procedures. Our protocol permitted a maximum interval of 6 months between APC surveillance and coiling catheterization procedures.

**Fig. 5:**
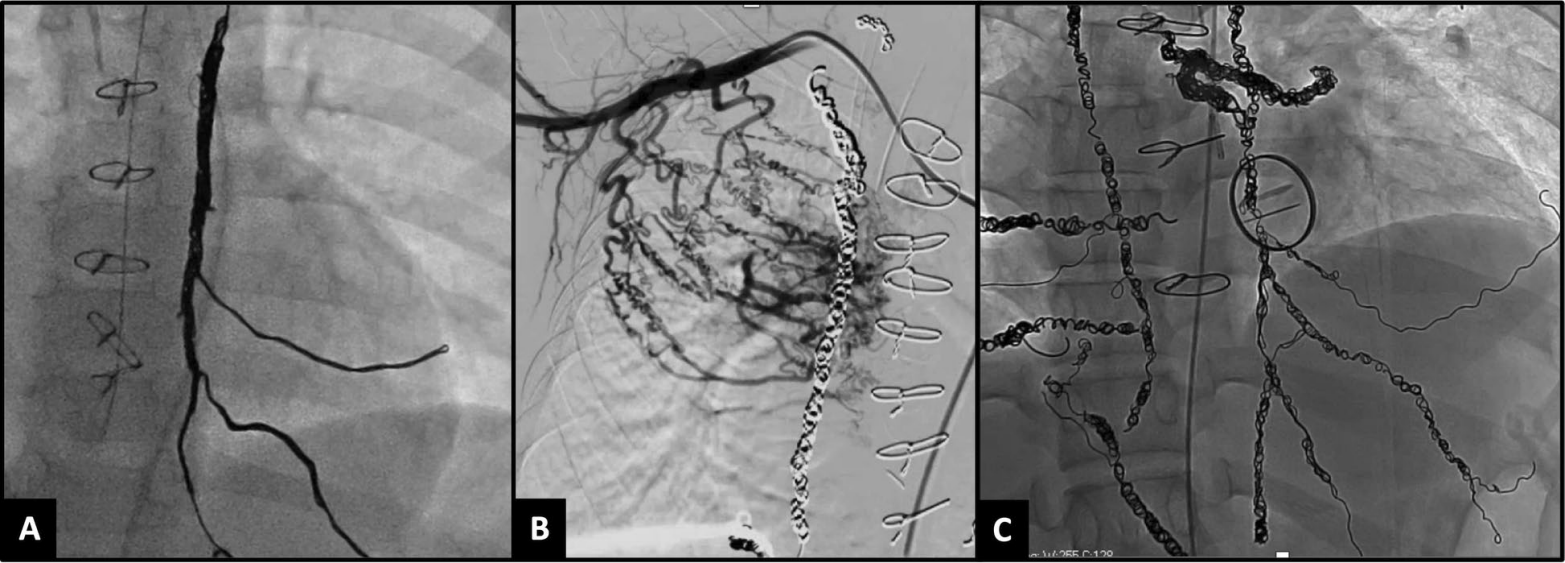
**A** It is important to occlude the internal mammary artery and its branches and anastomoses to the superior epigastric artery and intercostal arteries to prevent newer APC formation. **B** Coiling the internal mammary arteries can trigger the emergence of accessory mammary arteries from the lateral thoracic artery, thoracodorsal artery, and the pectoral branches of the thoracoacromial artery, as well as from the superior thoracic artery. **C** It is important to occlude the internal mammary artery and its branches and anastomoses to the superior epigastric artery and intercostal arteries to prevent newer APC formation

**Fig. 6:**
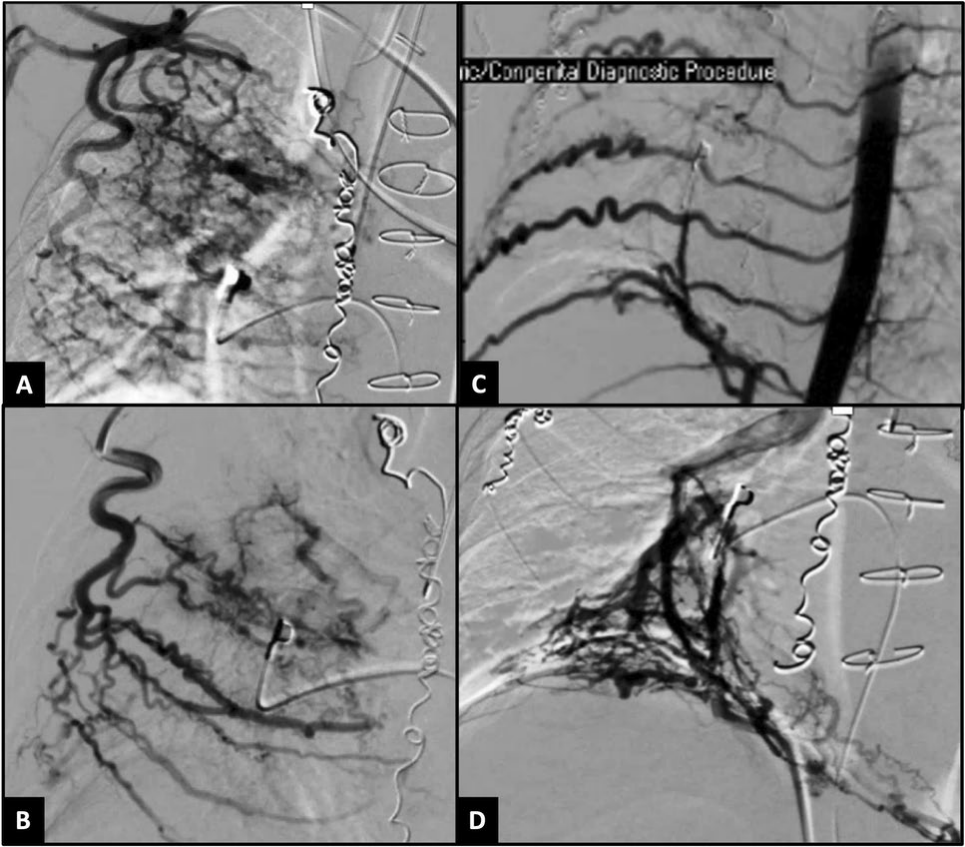
Coiling the internal mammary arteries can trigger the emergence of accessory mammary arteries from the lateral thoracic artery (**A**), thoracodorsal artery (**B**), and the pectoral branches of the thoracoacromial artery (**A**), as well as from the superior thoracic artery (**A**). These arteries connect with branches of the intercostal arteries (**C**), the superior epigastric artery, and the inferior phrenic artery (**D**). All these DSAs are from a 3-year-old patient with hypoplastic left heart syndrome post-Fontan, listed for heart transplantation

A network of vertical and horizontal arteries encircles the thoracic cavity, serving as a source for APCs. Numerous anastomoses exist between these arteries, meaning that coiling one vessel will enlarge anastomoses in others due to increased pulmonary steal through the APCs. For instance, coiling the internal mammary arteries can trigger the emergence of accessory mammary arteries from the lateral thoracic artery, thoracodorsal artery, and the pectoral branches of the thoracoacromial artery, as well as from the superior thoracic artery (Figs. 5 and 6). These arteries connect with branches of the intercostal arteries, the superior epigastric artery, and the inferior phrenic artery, which can have a variable origin—either directly from the aorta, celiac trunk, or occasionally the renal or suprarenal arteries (Fig. 6). As these anastomoses form, they contribute to additional APCs, due to the lower resistance to flow into the pulmonary circulation. Over time, these vessels enlarge, increasing the shunt volume and the pulmonary venous return to the heart, thereby progressively increasing the volume load on the single ventricle. If these vessels remain patent, the heart struggles to cope with the postoperative volume load on the ventricle. Similarly, the post-transplanted ventricle, which is usually stiffer, finds it challenging to manage the excess volume from the APC shunt return, leading to hemodynamic instability for patients over several days. While partaking in such an aggressive coiling strategy, close attention must be given to the origins of the anterior spinal arteries and the artery of Adamkiewicz, which can vary; occluding blood flow to these vessels risks permanent neurological damage.

**Fig. 7:**
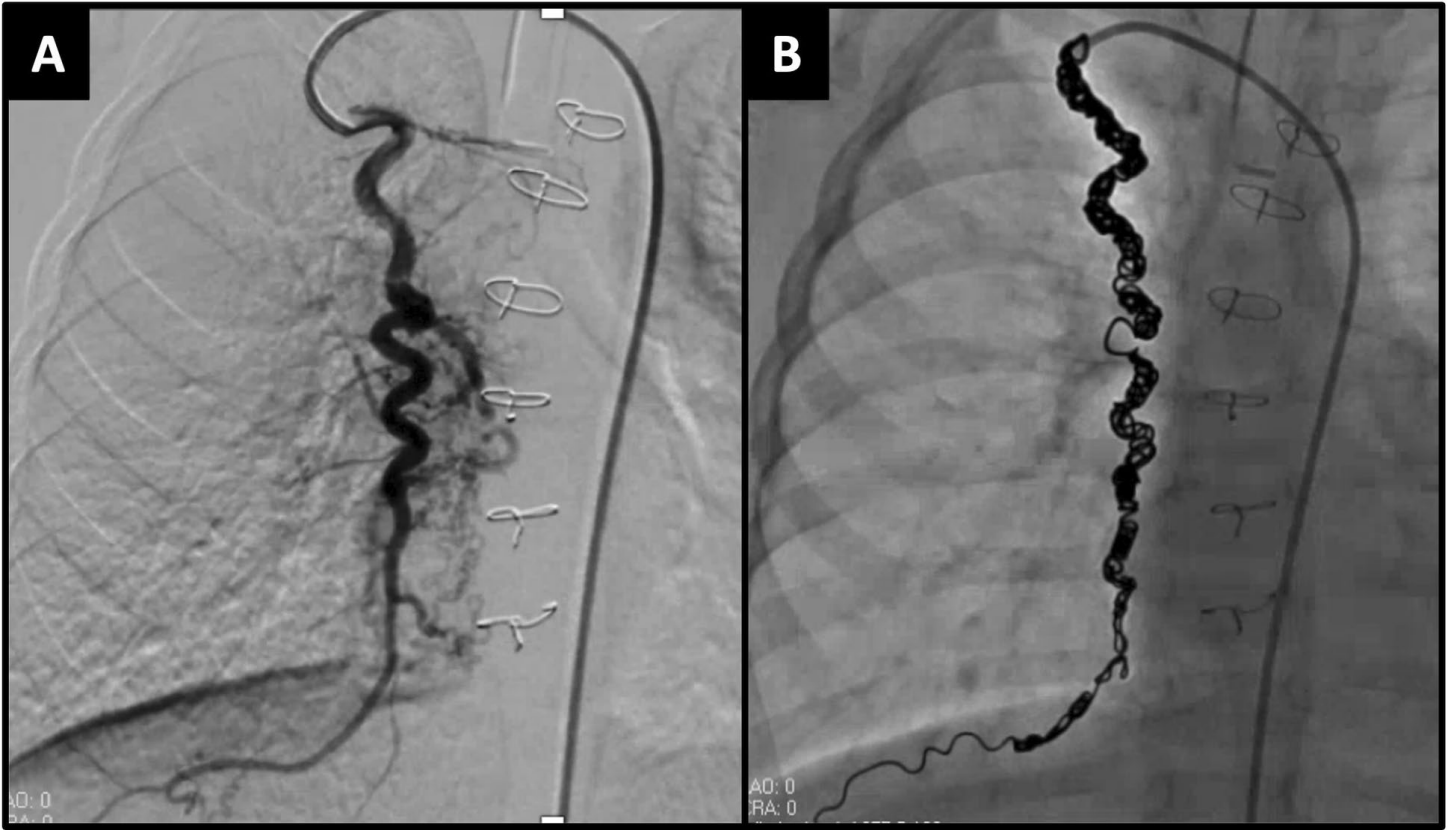
The Penumbra packing coil LP can take the shape of the blood vessels that they are introduced into even when they are highly tortuous as demonstrated in the pre (**A**) and post (**B**) coil embolization figures of the right internal mammary artery in this 2-year-old patient with pulmonary atresia with intact ventricular septum before the Fontan operation

**Fig. 8:**
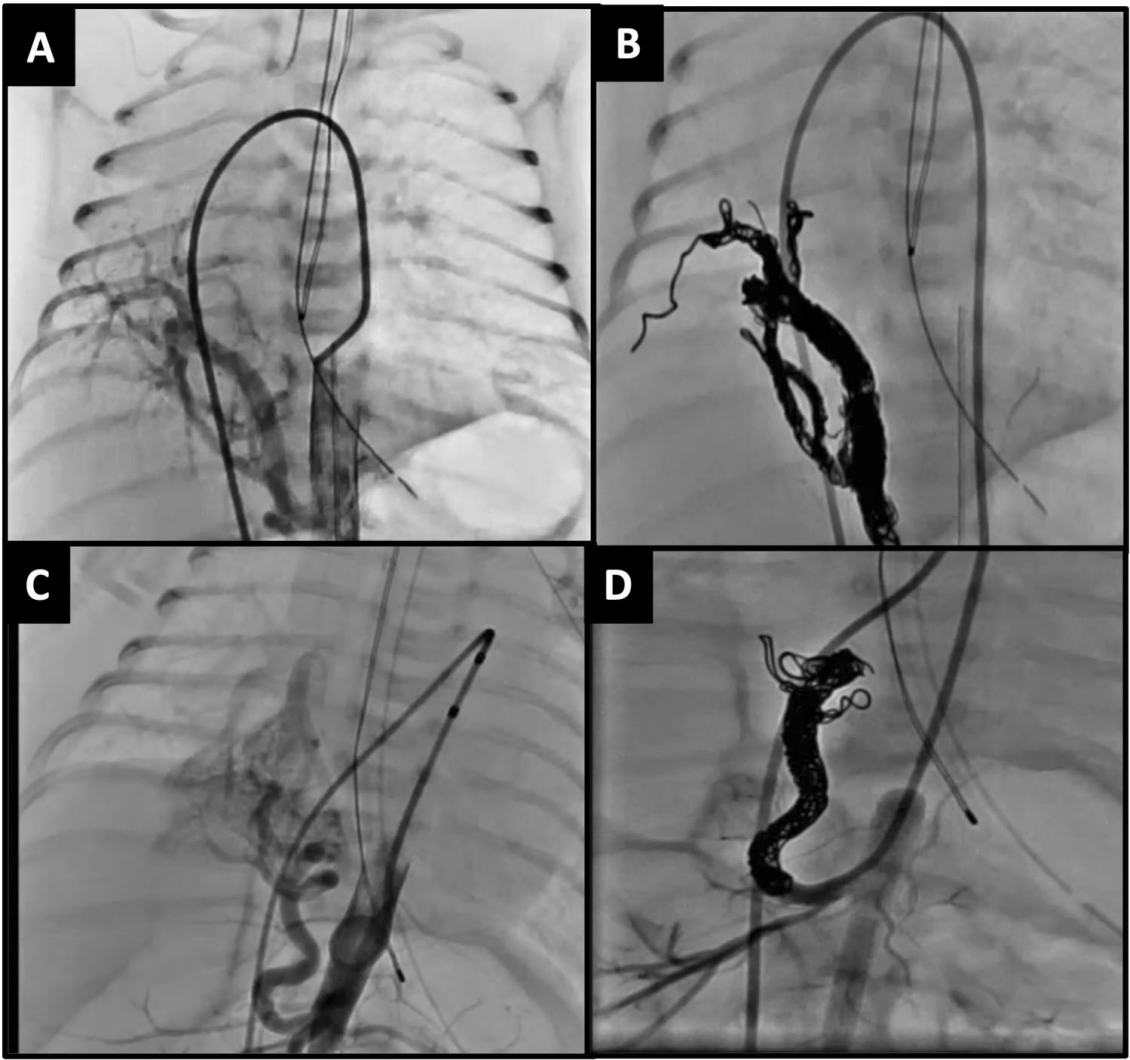
The low-profile packing coils are especially beneficial in these extremely small patients with Scimitar syndrome to occlude the afferent artery of extra-lobar pulmonary sequestration that carries a high mortality rate. The first patient is a 1.4 kg premature neonate (**A**) who underwent coil embolization using the Penumbra LP packing coil through a femoral venous approach to get a catheter through the right heart and across a patent ductus arteriosus into the aorta and the celiac trunk to occlude the aberrant vessel without blocking blood flow through other branches of the celiac artery (**B**). The second patient is a 1.7 kg premature neonate (**C**) who underwent the same technique to occlude the aberrant vessel without blocking blood flow through other branches of the celiac artery (**D**) using the Penumbra LP packing coil

**Fig. 9:**
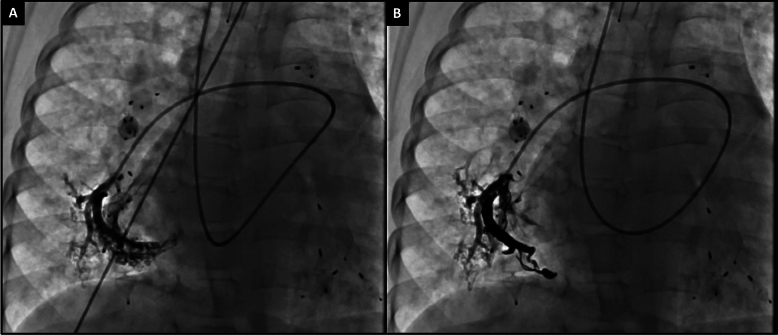
The packing coils are also extremely useful to completely occlude pulmonary arteriovenous malformation (PAVM) as demonstrated in these pre (**A**) and post (**B**) occlusion images. This is a 12-year-old patient with hereditary hemorrhagic telangiectasia with multiple PAVMs that were difficult to treat with vascular plugs alone in the past. With the availability of the Penumbra packing coils, the entire PAVM can be completely occluded with no residual shunting

Penumbra’s Packing Coils (Fig. 2) are designed to densely fill vessels, achieving swift and dependable occlusion. We have found that when APC’s are not occluded throughout the length of the entire vessel, smaller APC’s can develop distally despite proximal occlusion because of their communication with other arteries and failure of complete thrombus formation within the vessel. Because we rely on complete APC occlusion as distal as possible to prevent this APC recurrence, the “liquid metal” design offered by the Packing coils enables them to conform to any vessel diameter, ensuring complete and immediate occlusion while reducing reliance on thrombus formation (Fig. 7). In neonatal applications, the Ruby LP and Packing Coil LPs are specifically designed for small vessels, allowing interventionists to navigate challenging anatomies even in neonates. These coils have demonstrated effectiveness in treating conditions like Scimitar syndrome (Fig. 8), pulmonary arteriovenous malformations (Fig. 9), where control and minimized procedural invasiveness are critical. Penumbra Coils are also fully retractable, which we have found enhances control during deployment. This ensures precise placement, shortens procedural time, and decreases complications such as coil migration or incomplete occlusion.

## Conclusions

Applying APC coiling before the Fontan procedure has been observed to decrease chest tube output and reduce the number of postoperative hospitalization days. Furthermore, it may improve the 1-year survival rate following heart transplantation in pediatric patients experiencing Fontan failure when the APCs are pre-coiled. The Penumbra packing coils and Ruby coils are recognized for their significant efficacy in managing APCs in the pediatric population.

## Data Availability

No datasets were generated or analysed during the current study.
